# Noninvasive blood glucose sensing by secondary speckle pattern artificial intelligence analyses

**DOI:** 10.1117/1.JBO.28.8.087001

**Published:** 2023-08-01

**Authors:** Deep Pal, Amitesh Kumar, Nave Avraham, Yoram Eisenbach, Yevgeny Beiderman, Sergey Agdarov, Yafim Beiderman, Zeev Zalevsky

**Affiliations:** a Bar-Ilan University, Faculty of Engineering, Ramat Gan, Israel; b Indian Institute of Technology (Indian School of Mines) Dhanbad, Department of Electronics Engineering, Dhanbad, Jharkhand, India; c Holon Institute of Technology, Faculty of Electrical Engineering, Holon, Israel

**Keywords:** artificial intelligence, blood glucose detection, deep neural networks, magneto-optics, noncontact speckle-based measurement, noninvasive

## Abstract

**Significance:**

Diabetes is a prevalent disease worldwide that can cause severe health problems. Accurate blood glucose detection is crucial for diabetes management, and noninvasive methods can be more convenient and less painful than traditional finger-prick methods.

**Aim:**

We aim to report a noncontact speckle-based blood glucose measurement system that utilizes artificial intelligence (AI) data processing to improve glucose detection accuracy. The study also explores the influence of an alternating current (AC) induced magnetic field on the sensitivity and selectivity of blood glucose detection.

**Approach:**

The proposed blood glucose sensor consists of a digital camera, an AC-generated magnetic field source, a laser illuminating the subject’s finger, and a computer. A magnetic field is applied to the finger, and a camera records the speckle patterns generated by the laser light reflected from the finger. The acquired video data are preprocessed for machine learning (ML) and deep neural networks (DNNs) to classify blood plasma glucose levels. The standard finger-prick method is used as a reference for blood glucose level classification.

**Results:**

The study found that the noncontact speckle-based blood glucose measurement system with AI data processing allows for the detection of blood plasma glucose levels with high accuracy. The ML approach gives better results than the tested DNNs as the proposed data preprocessing is highly selective and efficient.

**Conclusions:**

The proposed noncontact blood glucose sensing mechanism utilizing AI data processing and a magnetic field can potentially improve glucose detection accuracy, making it more convenient and less painful for patients. The system also allows for inexpensive blood glucose sensing mechanisms and fast blood glucose screening. The results suggest that noninvasive methods can improve blood glucose detection accuracy, which can have significant implications for diabetes management. Investigations involving representative sampling data, including subjects of different ages, gender, race, and health status, could allow for further improvement.

## 1 Introduction

Glucose is one of the essential molecules that creates energy in the human body. Glycolysis is a metabolic pathway that breaks down glucose to pyruvic acid, releasing energy. Glucose is transported from the small intestine to the villi and into the bloodstream. Oxygen and glucose are carried by the bloodstream and taken up by the bodily cells. Insulin is a hormone that moves glucose into the cells for the metabolic pathway.^1^^,^^2^ A high glucose level in the blood is a metabolic disease called diabetes mellitus. Diabetes patients must regularly check their blood glucose levels to remain within a healthy range. The fast and efficient evaluation of blood glucose concentration requires the development of new measurement methods for which accuracy, dependability, high sensitivity, quick reaction, cheap cost per test, mobility, and a noninvasive procedure are significant factors.^2^

The initial technique for measuring blood glucose concentration depends on glucose’s capacity to function as a reductant in the copper iodine solution, known as the “copper-iodometric method.” The “enzymatic method” is a different technique for measuring glucose. The catalytic activity of the enzymes is a critical component of enzyme-based glucose detection techniques. From the fundamentals of the “copper-iodometric method,” “non-enzymatic glucose sensors” containing metal electrodes were later developed. High-performance liquid chromatography is also a helpful tool for separating and identifying chemical components of organic material, including glucose. The current method of measuring blood glucose involves skin-punching a finger to extract a blood droplet that is then placed on a strip and into a glucometer. All of these methods require blood extraction, which is painful and time-consuming.^1^^–^^3^

The advantage of the noninvasive methods is that puncturing the skin and drawing blood, which causes pain or trauma to the patient, is not required. Over time, several noninvasive techniques have been developed, as shown in Table 1. Transdermal measurements are noninvasive techniques for evaluating blood glucose. These technique involve using chemicals, electricity, or ultrasound to draw glucose through the interstitial fluid. The most noninvasive glucose monitoring techniques are made to identify the optical signature of blood glucose in which the light focused on biological tissue either transmits, scatters, or reflects depending on the sample’s structural and chemical components.

**Table 1:** Comparison of non-invasive techniques for blood glucose monitoring.

Technique	Methods used	Comments	References
NIRS	Transmittance, reflectance, and diffuse reflectance	Fast, accurate, and widely available. Sensitive to interference from ambient light, affected by skin pigmentation, and limited penetration depth.	4 and 5
Raman spectroscopy	Raman scattering and surface-enhanced Raman scattering (SERS)	Accurate and able to distinguish glucose from other analytes. Limited penetration depth and requires expensive equipment.	4 and 6
Fluorescence spectroscopy	Intrinsic and extrinsic fluorescence, time-resolved fluorescence	Sensitive and able to distinguish glucose from other analytes. Limited penetration depth and may cause tissue damage.	4 and 6
OCT	OCT	High resolution and able to measure glucose in specific tissue layers. Limited penetration depth, expensive, and requires specialized equipment.	4 and 6
PAS	Photoacoustic effect	High sensitivity and able to measure glucose in specific tissue layers. Limited penetration depth requires expensive equipment.	4 and 6
Impedance spectroscopy	Electrical impedance spectroscopy (EIS), bioimpedance analysis (BIA)	Low cost and able to measure glucose continuously. Affected by temperature and humidity and may require frequent calibration.	4 and 7
Microwave spectroscopy	Microwaves	Able to measure glucose continuously. Affected by changes in tissue water content and limited penetration depth.	4 and 8
Thermography	Thermal imaging	Low cost and able to measure glucose continuously. Affected by changes in skin temperature and limited accuracy.	4 and 9
Sweat-based glucose monitoring	Sweat analysis	Continuous measurement possible and low-cost. Affected by skin temperature, sweat rate, and sensitivity may not be high enough for some applications.	4 and 6

Table 1 provides an overview of several non-invasive techniques for measuring blood glucose levels. Each technique has its own strengths and limitations. Near-infrared spectroscopy (NIRS) and mid-IR (MIR) are the most common noninvasive glucose measurement methods. Near-infrared light scatters less than ultraviolet or visible light. It can be detected and measured using reflection and transmission and has a relatively high capacity to enter bio fluids and soft tissues.^4^^,^^6^ NIRS utilizes the focused laser beam to determine glucose concentration in the tissues by tracking variations in light intensity produced by transmission and reflection in the tissue.^7^ The method has significant limitations affecting physicochemical characteristics, including body temperature, skin pigmentation, and blood pressure variations. Blood glucose levels and NIR measurements from the finger are correlated, but the clinical acceptability of these measurements was unsatisfactory. Higher wavelengths used in MIR spectroscopy have a reduced scattering and higher absorption. More distinct MIR spectral bands compared with NIR are generated by glucose. However, due to extremely low penetration and selectivity, light encounters the same limitations as NIR because it only penetrates the skin for a few millimeters.^4^^,^^7^

Raman spectroscopy is also considered one of the most effective techniques for measuring blood glucose levels. The Raman method uses a monochromatic light source that ranges from visible to MIR based on the Raman effect. A tissue sample illuminated by monochromatic light results in scattered rays flowing in all directions. Most of the rays have a wavelength similar to incident light. The interaction of the remaining beams with the tissue sample, which causes rotation and vibration, results in inelastic scattering, also known as Raman scattering, with wavelengths distinct from the input light. The Raman shift is the resultant difference in the wavelength reflecting bodily fluids’ rotational and vibrational states. Raman spectroscopy has many applications, including a higher penetration depth than MIR and high specificity. However, a longer spectrum capture time is necessary, and the laser intensity and wavelength instability also impact the measurements.^4^^,^^6^^,^^10^

Instead of directly detecting glucose, fluorescent sensing technology analyzes the signals of molecules that can reversibly mix with glucose. Because glucose molecules’ fluorescence is too weak, it can easily interfere with the direct fluorescent properties. As a result, most studies concentrate on indirect fluorescent labeling, which involves adding a fluorophore to bind to glucose molecules. Only then will fluorescence be emitted, indicating the presence of glucose molecules. This method makes measurements possible, and changes in light intensity do not affect the signal. However, several factors, such as skin pigmentation, degree of erythema, and epidermis layer thickness, affect the fluorescence intensity.^6^^,^^10^ Optical coherence tomography (OCT) has a high resolution and can measure glucose in specific tissue layers, but it has a limited penetration depth and requires specialized equipment. Photo acoustic spectroscopy (PAS) is highly sensitive and measure glucose in specific tissue layers, but it has a limited penetration depth and requires expensive equipment. Impedance spectroscopy is low cost and measures glucose continuously, but it is affected by temperature and humidity and requires frequent calibration. Microwave spectroscopy and thermography are able to measure glucose continuously, but they are affected by changes in tissue water content and skin temperature, respectively. Sweat-based glucose monitoring allows for continuous measurement; it is affected by skin temperature and sweat rate, and its sensitivity may not be high enough for some applications. None of these methods have produced a device equivalent in its features to the current invasive device. Some methods were only tested in the laboratory using glucose solution samples and have not been tested *in-vivo*.^4^^–^^8^^,^^10^^–^^13^

A new noninvasive method remotely monitors medical parameters using temporal analysis of secondary speckle patterns generated by reflected light from the human skin illuminated by a laser beam. Numerous biomedical measurements techniques have already been successfully developed using this method, such as blood pulse pressure, heart rate, breathing rate, blood coagulation, blood oxygen saturation, intraocular pressure, and glucose measurements.^2^^,^^3^^,^^9^^,^^13^^–^^16^ Our previous work proposes a non-invasive technique for sensing glucose concentration in the bloodstream using a multimode fiber-based sensor. The multimode fiber (covered and uncovered) touching the subject’s finger under a magnetic field is applied to detect the glucose concentration. The uncovered fiber placed below the finger under an alternating current (AC) magnetic field (150 Gauss) at 140 Hz was found to have a lock-in amplification role, improving glucose sensing.^17^ The current work further develops the speckle-based noninvasive blood glucose sensing combined with data processing by machine learning (ML) and deep neural network (DNN) analysis. The AC-induced magnetic field at a particular frequency improved the selectivity of blood glucose detection. The recorded secondary speckle pattern data are preprocessed and modified to acquire the lock-in amplification in glucose data at a particular frequency of the applied magnetic field. ML and DNN analysis improve the accuracy of blood glucose sensing. Although the present study achieved high accuracy, the experimental system still requires modifications for onsite or online monitoring of glucose levels. Other researchers have explored more integrated systems, such as planar waveguide sensors^18^ and evanescent waveguide sensors,^19^ which may offer improved sensitivity and selectivity. However, the mentioned sensors have not been configured for the field application or tested *in vivo*. Evaluating glucose concentration indicates a possible application for diabetes monitoring. To allow for the onsite/online monitoring, we are planning to build a compact hand bracelet containing a magnetic field source, laser, and a camera for non-invasive blood glucose sensing. The bracelet will connect Bluetooth with a cellular phone for data recording and online transmission.

## 2 Theoretical Background

### 2.1 Speckle Pattern Analyses

Secondary speckle patterns are self-interfered random patterns created by a laser-induced coherent light reflected from a rough surface. Speckles can be a point of reference to monitor changes in the scattered light’s phase where the low-coherence light with different wavelengths is considered for analysis. In that situation, a speckle pattern will not typically be seen because the speckle patterns created by different wavelengths normally average each other out, which his known as self-interference.^16^^,^^17^

In our proposed sensing mechanism, the secondary speckle patterns reflected from a human skin illuminated by coherent light are captured using a defocused camera for temporal analysis. Instead of changing randomly while the camera is defocused, a speckle pattern will move or vibrate in the transversal plane. The speckle pattern is randomly varied because there are three types of movement co-occurring: transverse, axial, and tilt. Those three types of movement cannot be separated in a soft medium (such as a tissue). After making the necessary approximations, it was considered that the two types created hardly any differences in the acquired speckle pattern. The third type is the object’s tilting, which is expressed as shifts in the pattern of speckles, as we can see in the light distribution.^13^^,^^19^^,^^20^

Therefore, the overall effect of the three types of movement is only a shift in the transversal plane. The speckle patterns’ temporal trajectories are proportional to the signals to be extracted, the frequent change in the blood vessels, and the interaction between the coherent light and the illuminated surface, such as human skin affecting recorded speckle patterns.^13^^,^^15^^,^^16^^,^^20^

### 2.2 Speckle-Based Blood Glucose Sensing

Blood has four main components: plasma, red blood cells (RBCs), white blood cells, and platelets. Blood has many different functions, including transporting oxygen and nutrients to the lungs and tissues and forming blood clots to prevent excess blood loss.

Blood plasma occupies around 50% of the blood volume, containing 92% water, 7% proteins, 0.5% inorganic salts, and 0.07% to 0.1% glucose. It mainly comprises coagulants, fibrinogen and aid in blood clotting. Plasma proteins such as albumin and globulin help maintain the colloidal osmotic pressure at about 25 mmHg. The red blood cells - erythrocytes (RBCs) consist of hemoglobin (Hb), the iron-binding proteins in the Fe 3+ state, and are responsible for oxygen transfer. Water in the plasma consumes a considerable portion of the transmitted light compared with additional components. Glucose absorbed into the blood is found in the blood plasma and is combined with erythrocytes. Blood glucose is mainly controlled by evaluating its concentration in plasma, which is food intake dependent and undergoes fast temporal variation. Glucose dissolved in the blood plasma also binds to Hb in RBCs, forming glycosylated hemoglobin (HbA1c), which remains stable in the RBCs for three months and is an indicator of average blood glucose concentration over that period.^21^^,^^22^

When a laser beam illuminates the human skin, it is reflected from the surface while partially penetrating through the skin barrier and interacting with the blood and its components, including plasma and Hb. Therefore, the captured speckle patterns using the defocused camera could reflect the blood characteristics, including the glucose concentration.

The magneto-optic effect that Faraday first observed is used to measure changes in the optical properties caused by the concentration of glucose in the blood. Applying an AC magnetic field to the skin causes blood plasma glucose polarization, which affects the reflected secondary speckle patterns. The Faraday effect happens when linearly polarized light undergoes rotation when passing through a material medium in the presence of a magnetic field. This leads to circular birefringence, which causes the left and right waves to propagate at different rates due to the magnetic field’s influence. As a result, the speckle pattern changes as the polarization state of the wavefront changes, and this polarization state changes as the glucose concentration changes. Laser speckle imaging is used to measure the change in polarization properties and quantify blood glucose levels. In addition, the glucose concentration affects the blood pulse stream, including the tilt of the skin affecting the speckle patterns.^2^^,^^3^^,^^13^^,^^23^

A light beam’s polarization rotation angle φ changes as it passes through magneto-optic materials, and it is given as^14^^,^^22^

$$\varphi =\mathrm{VBL}=\frac{\pi LB\Delta n}{\lambda },$$ (1)

where V is the Verdet constant, B is magnetic field strength, L is the length of interaction, λ is the optical wavelength, and Δn is the rotation of light caused by the difference in the index of refraction between two circularly polarized states. Due to the low Verdet constant of most materials, weak magnetic fields only produce small rotations. Because of the large Verdet constant of the glucose molecule in comparison with molecules of other bloodstream components, we can assume that the interaction occurs primarily with the glucose dissolved in the plasma.^2^^,^^3^^,^^13^^,^^23^^,^^24^

The magnetic rotatory power is commonly called Verdet’s constant and is given as

$$V=\frac{\varphi }{LB\;\cos\;\alpha },$$ (2)

where φ is the rotation of the polarization plane, which occurs in a path of length L; B is the magnetic field’s strength; and α is the angle between B and the path of the light.^3^^,^^13^^,^^25^

For a given propagation distance L inside the medium, Eq. (1), which gives the change in the optical path, is used to calculate the minimal magnetic field intensity, causing decorrelation. As demonstrated before, the lowest magnetic field ($B_{\min}$) strength required to decorrelate the speckle area is proportional to the following:^2^

$$B_{\min}\propto \pi LR\varphi ,$$ (3)

where R is the illumination beam’s radius, which is a measure of the size of the laser beam. Equation (3) specifies the proposed method’s ability to detect magnetic fields.^2^^,^^13^^,^^23^

**Fig. 1:**
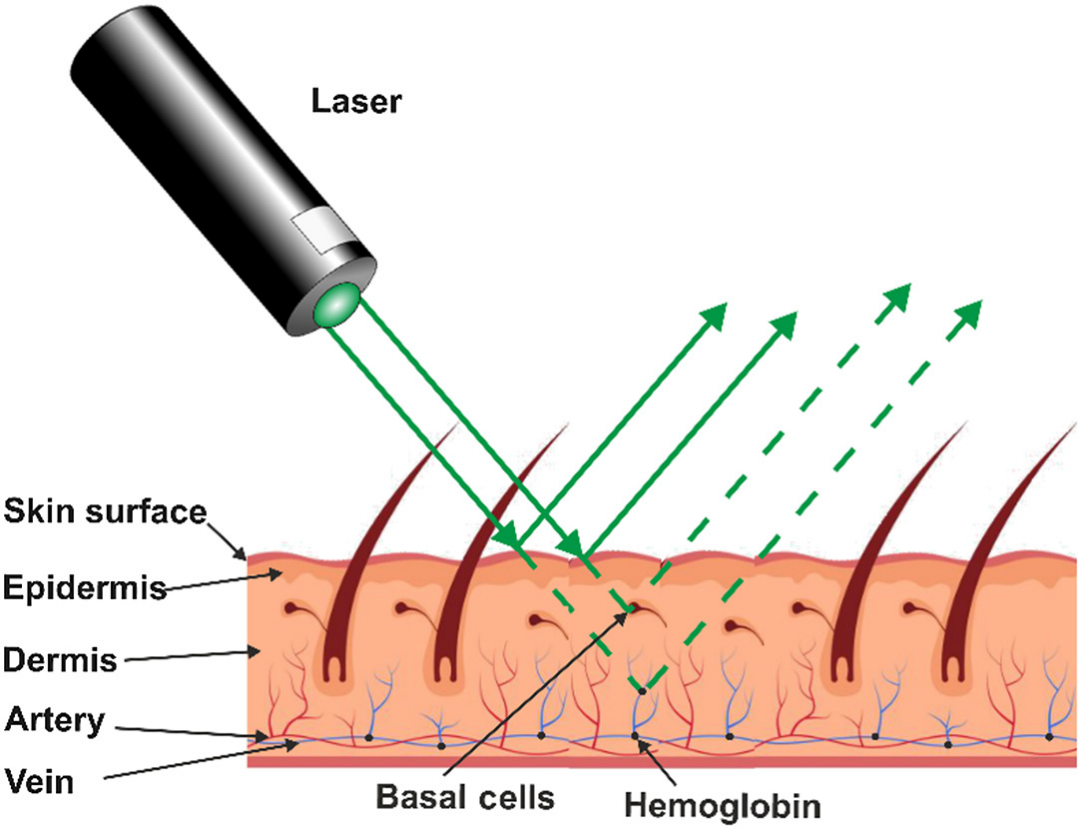
Skin-laser beam interaction.

The premise that the laser beam interacts with the blood’s glucose depends on the Beer-Lambert law. The human finger has three main layers: skin, adipose, and bone. In the adipose layer, blood vessels in the human finger variate in diameter (800  μm∼1.8  mm). The skin thickness is about 2 mm. The adipose layer between the skin and the blood vessel has a thickness of about 1-2 mm.^26^^,^^27^ The penetration of the laser into the finger interacts with the blood and gets reflected, showing the glucose profile, as demonstrated in Fig. 1.When the laser is positioned correctly, the light gets through only the skin and adipose in the worst scenario. Beer-Lambert’s law states that the attenuation of light is inversely related to the sample’s thickness and component concentration, given as

$$A=\ln(\frac{I}{I_{0}})=\varepsilon \times C\times d,$$ (4)

where A is the absorbance, ε is the coefficient of molar attenuation, C is the amount of the absorbent elements present, and d is the path length of light through the sample. I is the intensity of incident light, and $I_{0}$ is the transmitted light intensity. Absorbance usually refers to absorption, scattering, and reflection. Our system assumes the reflection after the light interaction with the blood vessels. Therefore, our path length is double.^24^^–^^26^^,^^28^

The noise overlapping the Faraday effect is reduced using the AC magnetic field applied at a fixed frequency (we used 140 Hz), creating a magneto-optic effect on blood glucose. The magneto-optic effect produces a lock-in amplification that makes it possible to monitor blood glucose levels precisely. If the acquired data are inferred at that frequency and analyzed using ML and DNNs, blood glucose detection can be highly selective and sensitive.^3^^,^^13^

However, the movement of Hb under the influence of a magnetic field also affects the recorded speckle patterns. When exposed to a magnetic field, Hb experiences a force due to its diamagnetic properties and resistance related to the plasma viscosity. This force causes the Hb molecules to oscillate within the plasma, altering the way in which light is transmitted. We also found reports that the blood plasma viscosity is slightly related to glucose concentration. Variation of blood plasma glucose from 100 to 400  mg/dl could increase the viscosity by 25%. Therefore iron-containing Hb, oscillating under the AC magnetic field within the plasma, could be affected depending on the applied frequency. It could also affect the recorded speckle patterns. The glycosylated Hb remains stable during the testing period and has a minimal effect on plasma blood sensing.^29^

## 3 Experimental Setup

**Fig. 2:**
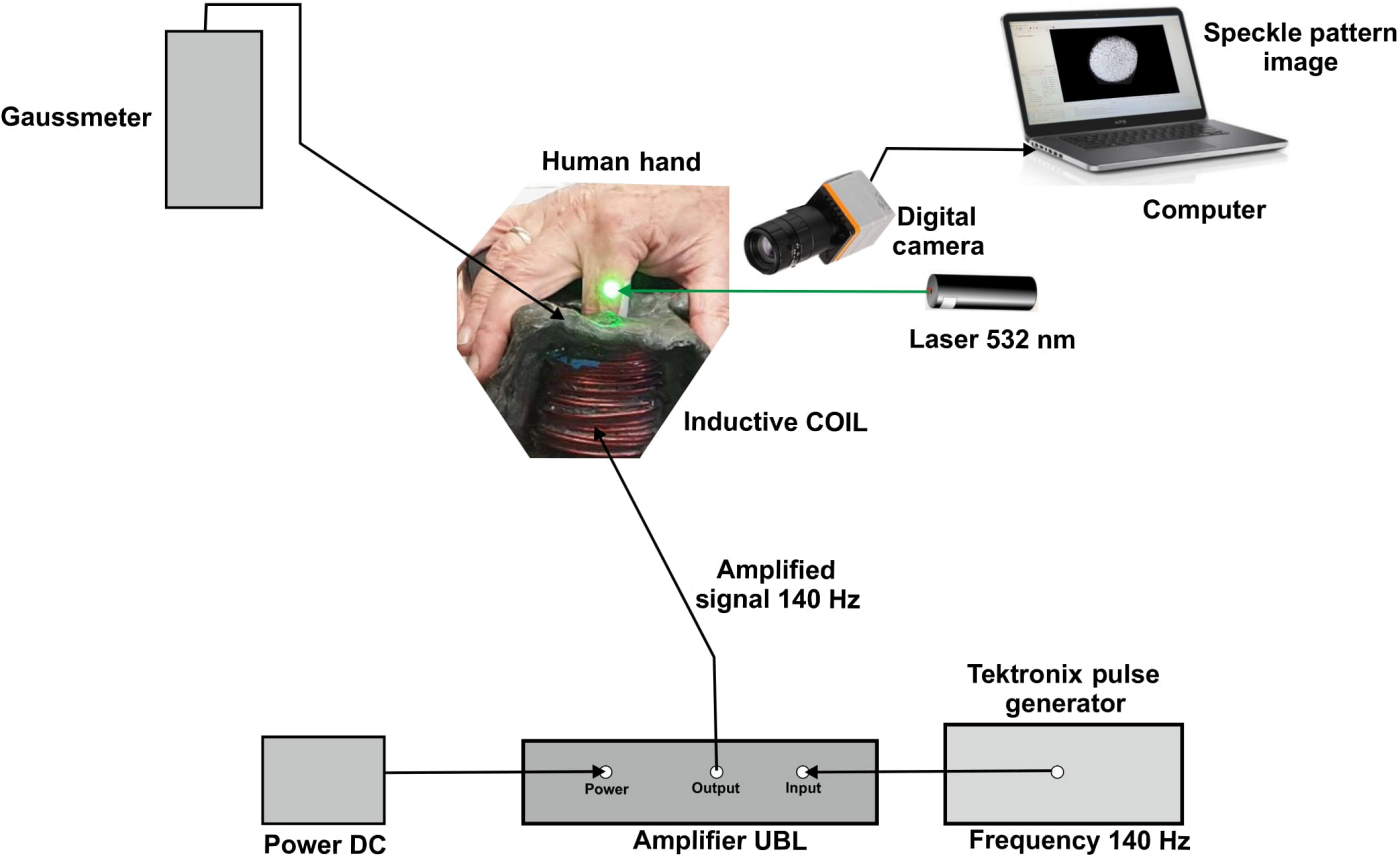
Schematic setup for noninvasive blood glucose sensing.

**Fig. 3:**
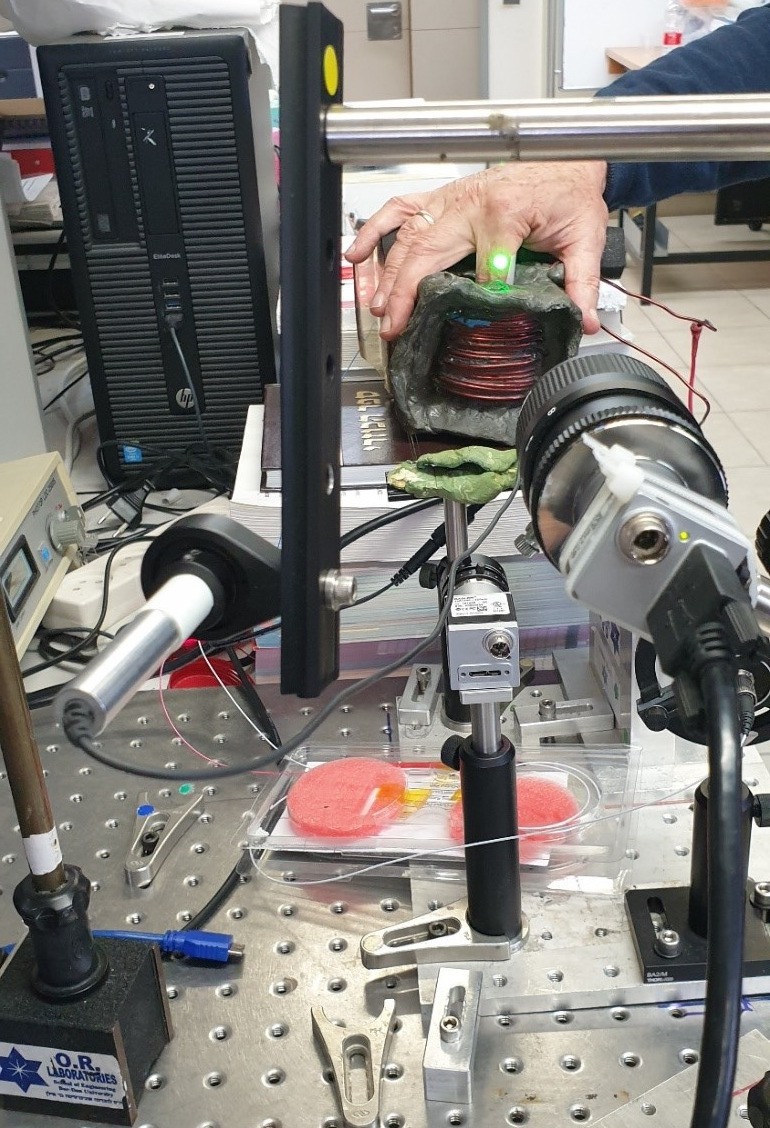
Experimental setup for blood glucose sensing.

A green laser (wavelength of 532 nm), a Basler camera with defocused optics, a magnetic field inductor, and a computer make up the setup for blood glucose measurement. The solenoid in the inductor is powered by a 12V DC battery supplying the UBL amplifier to boost the AC generated by the Tektronix pulse generator. The pulse generator’s frequency was set at 140 Hz. To store and process the speckle pattern recordings, the camera is connected to a computer. While the subject’s finger is partially inserted into the solenoid, the camera records the speckle pattern images reflected from a finger illuminated by a laser beam, at 500 frames per second (See Figs. 2 and 3).

Six healthy humans, ages 18 to 75, participated in our trial. Each participant was tested for two cases: (a) low glucose concentration after 12 hrs of fasting and (b) a series of relatively high glucose concentrations after a meal. The reference base true glucose levels were measured for each subject using the traditional finger-prick method before each recording of speckle patterns. To reduce motion artifacts in the glucose measurement, we stabilized the participants and setup and repeated the tests for five times, resulting in accurate measurements. The acquired speckle data were recorded under normal conditions and a magnetic field (150 Gauss) induced by 140 Hz AC to analyze the blood glucose concentration. A GM2 Gauss meter measured the magnetic field strength. To demonstrate the superiority of the magneto-optic effect over direct speckle pattern analysis, the data without a magnetic field were used as a baseline for performance comparison. This comparison highlighted the effectiveness of the magneto-optic effect in accurately differentiating glucose levels.

The recorded video data were preprocessed and modified to detect and classify blood glucose under normal and AC magnetic field conditions. The DNN data preprocessing is implemented by calculating the pixel-wise difference between two consecutive images. By contrast, ML algorithm data preprocessing is done by conversion of data in time series 2D movement using statistical methods. The preprocessed series of image input data for the DNN is normalized to make it optimal for a convolution-based network. The ML data are modified in time series form, which is further modified for the case of applied AC magnetic fields to infer data at an applied frequency.^17^ Each person was tested several times during each session to increase the variability in data.

## 4 Blood Glucose Concentration Prediction Models

Artificial intelligence (AI) for optical sensors has been increasingly common recently, particularly for tracking and boosting the accuracy of optical sensors for better performance. We modified and tested ML and DNNs models for blood glucose classification using the recorded data to find the best model. The recorded speckle pattern data are modified and preprocessed based on the model input type, which will be advantageous in detecting blood glucose accurately. In the case of DNNs, the input data, under normal conditions and an AC-induced magnetic field, is processed similarly, creating a modified image series input data, which is explained in Sec. 4.1. ML algorithms use input data in time series 2D form, implemented under normal conditions and and with an AC magnetic field applied. Further, with the influence of the AC magnetic field at the applied frequency (140 Hz), the magneto-optic effect creates a lock-in amplification that helps to precisely detect blood glucose. The process of converting recorded speckle images in time series and selecting the data only at 140 Hz is discussed in Sec. 4.2.

### 4.1 Data Processing for Deep Neural Networks

A component of AI techniques based on learning data features is deep learning. DNNs handle data in intricate ways using a sophisticated mathematical model. The input data used for training is modified to optimize the performance. The dataset of recorded videos was acquired and preprocessed for use in Matlab for training our model. Each acquired video, specified by its filename, represents a particular glucose level. The original data contain 162500 128×128 size frames; each video consists of 2501 frames recorded over 5 s. Each video has a distinct identity, including the glucose level under normal conditions with the AC magnetic field at 140 Hz and the actual reference blood glucose level. The images were taken from each video in the data preprocessing and saved as four-dimensional tensors (also known as multidimensional arrays) into a single Matlab file, which contains image data in rows to read data quickly and effectively.

**Fig. 4:**
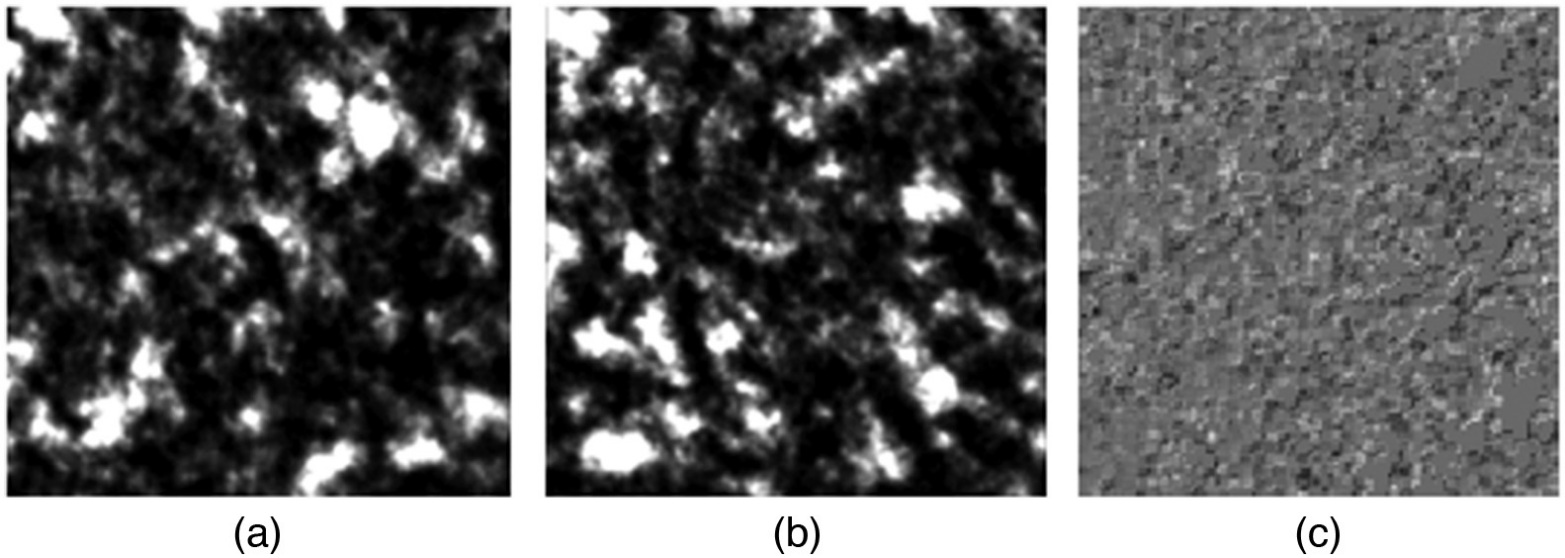
(a) and (b) Speckle patterns in two consecutive frames and (c) pixel-wise difference between two consecutive frames.

**Fig. 5:**
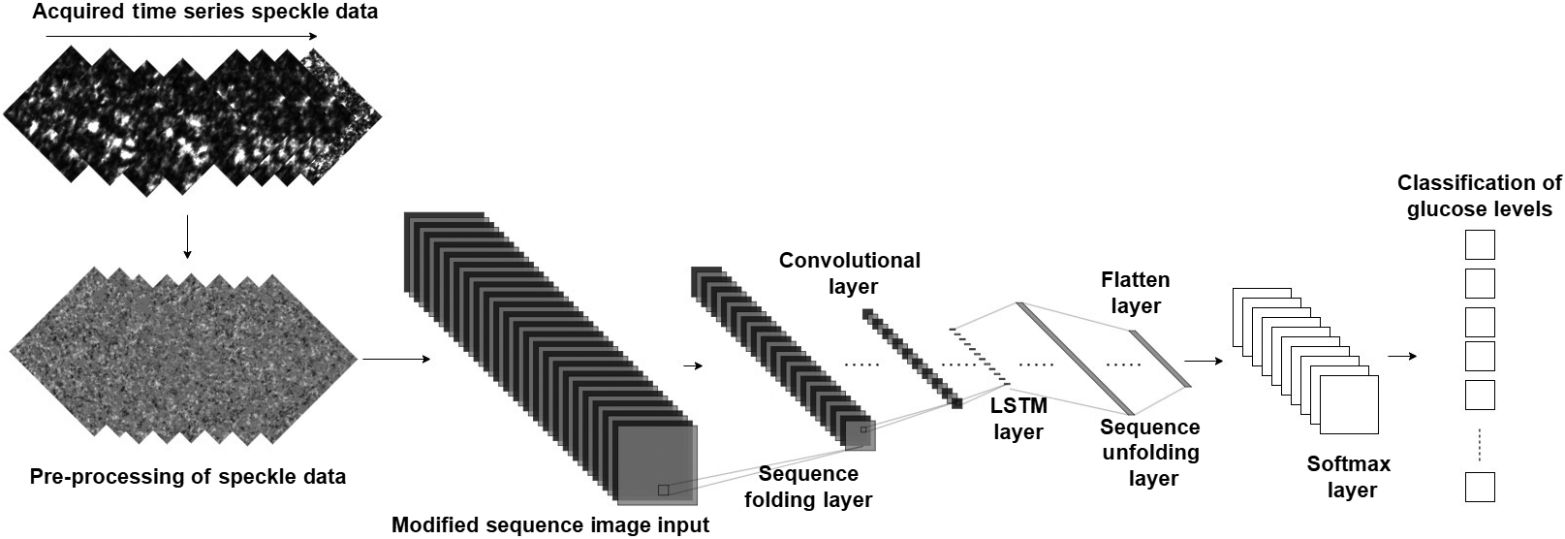
DNN blood glucose detection flow diagram.

To extract the temporal speckle pattern variation for different glucose levels, we use datastores, which act as a repository for data having the same structure and formatting. The pixel-wise difference between two subsequent images is calculated per video from datastores, as shown in Fig. 4. To further modify the input data suitable for the DNN model, the preprocessed images are normalized and scaled. The normalized data with the series of images indicating different glucose levels in the feature vector extracts features from each frame using a convolutional-based neural network (CNN). A CNN’s network architecture is improved and modified for optimal performance to produce the best results. Figure 5 presents the final network architecture representing the blood glucose detection flow chart. It uses the sequences to train a long short-term memory (LSTM) network to predict glucose levels.

In the learning process, the model generalizes the classification of glucose levels from the given dataset. First, the challenge of classifying optimally from the dataset is represented, so the model learns complex and accurate decision rules. To prevent overfitting, 30% of unused data is kept aside to check whether the model provides meaningful information and a precise decision. To prevent overfitting, dropout techniques, in which arbitrary units are thrown out throughout the training to prevent strong mutual dependencies between units, are used. This deep learning approach offers many benefits when dealing with series image input data. The model transforms a batch of image sequences into a batch of images by employing a sequence input layer, followed by a folding sequence layer. The convolutional layers extract features by calculating the dot product between the input image and each pixel entry making up the filter. Convolutional operations classify blood glucose levels, so each layer identifies unique visual elements from each frame. To categorize the obtained vector sequences and classify glucose levels, we included the LSTM layers followed by the output layers. A sequence unfolding layer and flattened layer were employed to recover the sequence structure and reformat the output into vector sequences. The DNN model is trained and optimized in Matlab, with an initial learning rate and gradient threshold of 0.0001 and 2, respectively. The mini-batch size was set at 16, and the data were shuffled at every epoch using the deep network designer that assisted in calculating N-dimensional arrays.

### 4.2 Data Processing for Machine Learning

ML algorithms were applied to classify blood glucose levels. The recorded video data contain temporal speckle pattern variations that need preprocessing and modification to extract features for classification using ML algorithms. The preprocessing is done using spatial pattern correlation analysis, which allows for tracking the relative shift of the speckle patterns of the two sequential frames to extract the total movement vector related to blood glucose concentration. The cross-correlation of the speckle pattern images is used to determine displacement across the X and Y axis. Each speckle image is correlated to the next image, and for each image, the correlation between the current and reference frames is calculated and averaged over time.^17^

The temporal variation of the graph peaks correlation value represents the changes in glucose levels. The data under normal conditions are directly used to train the algorithm. By contrast, the data under the 140 Hz AC-induced magnetic field are obtained using the fast Fourier transform (FFT) to transform the time series data into the frequency domain. By choosing our frequency of interest (140 Hz) from the FFT and using Matlab to eliminate any remaining frequency components, we can obtain the fluctuation only at 140 Hz. We used inverse FFT (IFFT) to recover time series data with variation only at 140 Hz.^17^ The obtained IFFT data comprise signals at a specific glucose level and are free from external noise. After being normalized, the preprocessed data are used as input to classify the glucose levels.

**Fig. 6:**
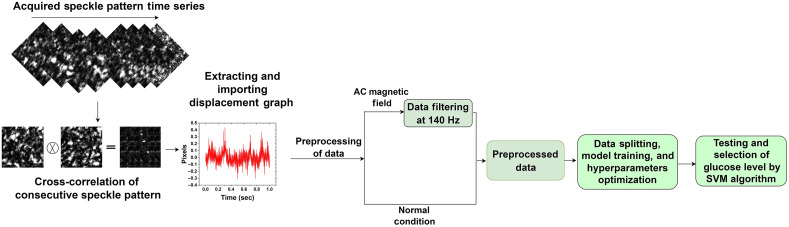
ML-based blood glucose detection by SVM algorithm – flow diagram.^17^

Each reference blood glucose level used in this study represents a vector with one row and 500 columns, ranging from 70 to 198  mg/dL. The preprocessed data are normalized and split for the training and testing by the selected algorithm. In a previous study,^9^ we found that the optimized support vector machine (SVM) algorithm performed better for AC-induced magnetic field data at 140 Hz than KNN, logistic regression, and decision trees. The model’s performance is improved by optimizing hyperparameters using a Bayesian optimizer. We implement five-fold cross-validation in which the dataset is randomly divided into five folds to prevent the algorithm from overfitting. Using the data from the first four folds, an ML model is trained, and its performance is analyzed on the validation set (fifth fold). The process is repeated until every five folds are implemented as a training set. The average prediction accuracy is calculated from the five different validation sets for each hyperparameter value. The best value for the hyperparameter is determined by analyzing the averaged prediction accuracy, resulting in increased model classification speed and improved model performance; see Fig. 6.

## 5 Results

Preliminary tests were conducted and analyzed to select the optimum frequency and magnetic field intensity for better glucose sensing. The glucose levels (normal and high) were analyzed under varying magnetic fields and operating AC frequencies. The 60, 90, and 140 Hz frequencies were analyzed using magnetic field strengths of 60, 120, and 150 Gauss. The magnetic field amplitude and frequency were selected by testing a range of frequencies that covered the available range of our setup. We observed that positive results were not obtained for 150 Gauss at 60 Hz, but we obtained significant results for 90 and 140 Hz. However, further analysis of 90 Hz and 140 Hz frequencies under 60 and 120 Gauss showed less accuracy than obtained under 150 Gauss. We concluded that higher magnetic field strength enhances the magneto-optic effect, leading to better glucose sensing. Moreover, we observed that 140 Hz gave better results than 90 Hz under a 150 Gauss magnetic field. Our primary aim was to demonstrate the possibility of detecting blood glucose by removing noise components using a particular frequency.

The analysis was done using an ML-based classification algorithm,^9^ explained in Sec. 4.2. The preliminary analysis gives the idea of selecting the optimum magnetic field of 150 Gauss and 140 Hz frequency. However, more advanced optimization analysis is required for better performance. After selecting the basic parameters, the experiments were conducted using our setup discussed in Sec. 3. Six healthy participants, ages 24, 25, 27, 28, 60, and 75, provided data for analysis. All glucose level detection experiments are performed in a controlled environment to achieve accurate, reproducible measurements and to minimize external environmental factors. The experiment involved measuring the subject’s blood glucose level using the traditional finger-prick method as a reference baseline, followed by testing with the optical setup. The tests were conducted in the morning after 12 hrs of fasting to standardize the low glucose level and then after a single meal to obtain glucose level variations over subsequent time intervals of 35 to 50 mins. Each participant was tested around 8 to 10 times, with 2-3 tests before and 6-7 tests after the meal to measure different glucose levels. To ensure accuracy, each measurement was repeated five times. The 13 glucose levels were selected from the combined measurements to reduce complexity and allow for a more focused and detailed analysis. This simplified the data analysis process, making it easier to draw accurate conclusions from the experiment. As illustrated in Fig. 2, the finger was partially placed into the solenoid, and the secondary speckle patterns were recorded to determine the blood glucose levels. The strength of the magnetic field was kept stationary (150 Gauss) and measured prior to every blood glucose level measurement. A shift of finger placement was performed for each reading to obtain variable data. The speckle patterns were recorded five times in a row for each glucose level. The subjects’ reference measurements are listed in Table 2. As explained, the measurement was done under normal and AC-generated magnetic fields.

**Table 2:** Reference blood glucose levels for the tested participants.

Sample No.	1	2	3	4	5	6	7	8	9	10	11	12	13
Glucose level (mg/dl)	86	89	92	93	96	105	113	125	135	137	146	177	198

The acquired data consist of 128*128  pixel images. Using MATLAB code, each speckle image is cross-correlated to the following frame for the DNN data input. The current and reference frame’s correlation was determined for ML and averaged across time, as explained in Sec. 4.

### 5.1 Evaluating Glucose Detection Accuracy Using a DNN

Our optimum network was tested using unseen data (testing data) gathered from the same six participants, and the data were not included during the training process. Table 2 provides the reference glucose level readings for each person evaluated. The data were split randomly with 70% of the videos used for training and the remaining 30% used for testing. Each recording was analyzed under a 140 Hz AC-induced magnetic field and without it (the normal condition). The accuracy of the DNN networks was tested, and finally, the CNN model architecture was selected, as shown in Fig. 5.

**Fig. 7:**
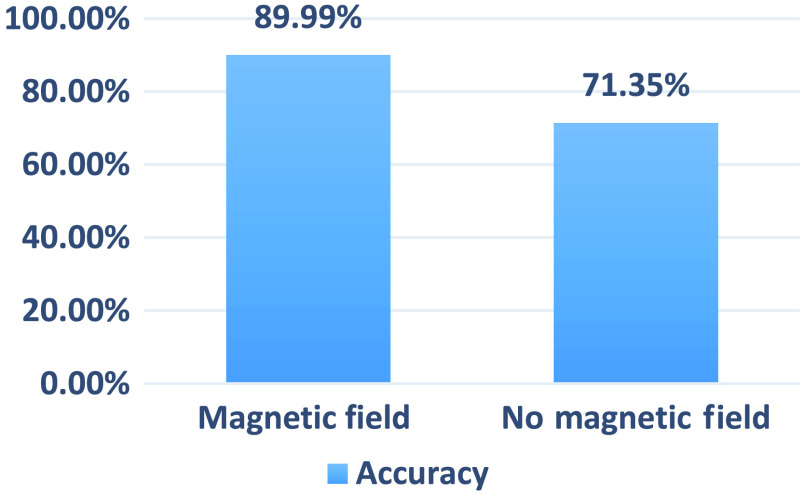
Accuracy of blood glucose classification using CNN.

**Fig. 8:**
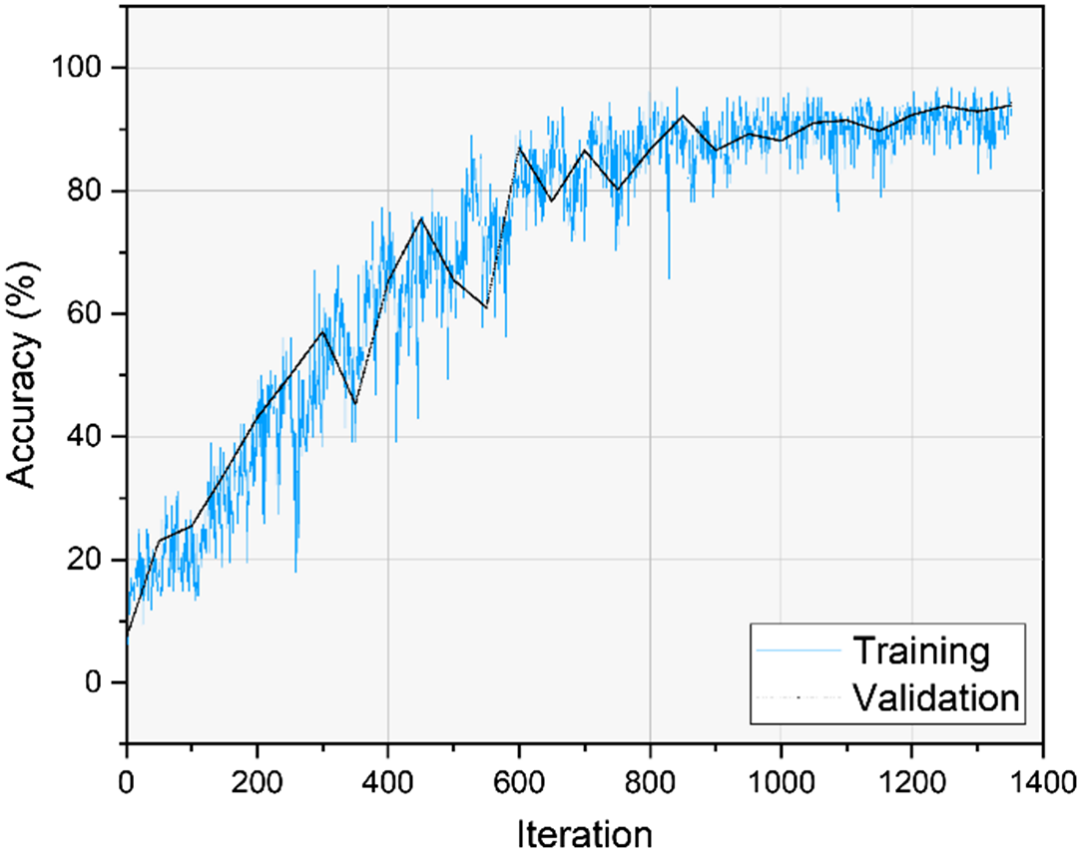
Training process of optimized CNN.

As seen in Table 3, other critical performance metrics of our network, such as accuracy, sensitivity, and F1 score, are computed. The F1 score represents the standard measure to rate a network’s success using each class’s weighted average of precision and recall using the following equations:

$$F1=2\times \frac{\text{precision}\times \text{recall}}{\text{precision}+\text{recall}},$$ (5)

where:

$$\text{Precision}=\frac{\mathrm{TP}}{\mathrm{TP}+\mathrm{FP}},\;\text{Recall}=\frac{\mathrm{TP}}{\mathrm{TP}+\mathrm{FN}},$$ (6)

where TP, FP, and FN are true positive, false positive, and false negative, respectively. The average F1 score of 0.8935 is calculated for assessing the network glucose prediction performance. CNN shows higher accuracy and the ability to identify various glucose levels. The optimized model that we achieved gives an accuracy of 89.9% on the testing dataset, with a magnetic field (150 Gauss) at 140 Hz, as shown in Fig. 7. Figure 8 represents the training procedure for our CNN. For training preprocessed images in a network, each image was treated individually, which means other images coming after or before or even belonging to the same recorded video do not affect the network performance.

**Table 3:** CNN model processing results.

Configurations	Accuracy	Precision	Sensitivity	F1 score
AC-induced magnetic field	89.99%	0.9064	0.8932	0.8935
Normal condition	71.35%	0.7689	0.7431	0.7350

**Fig. 9:**
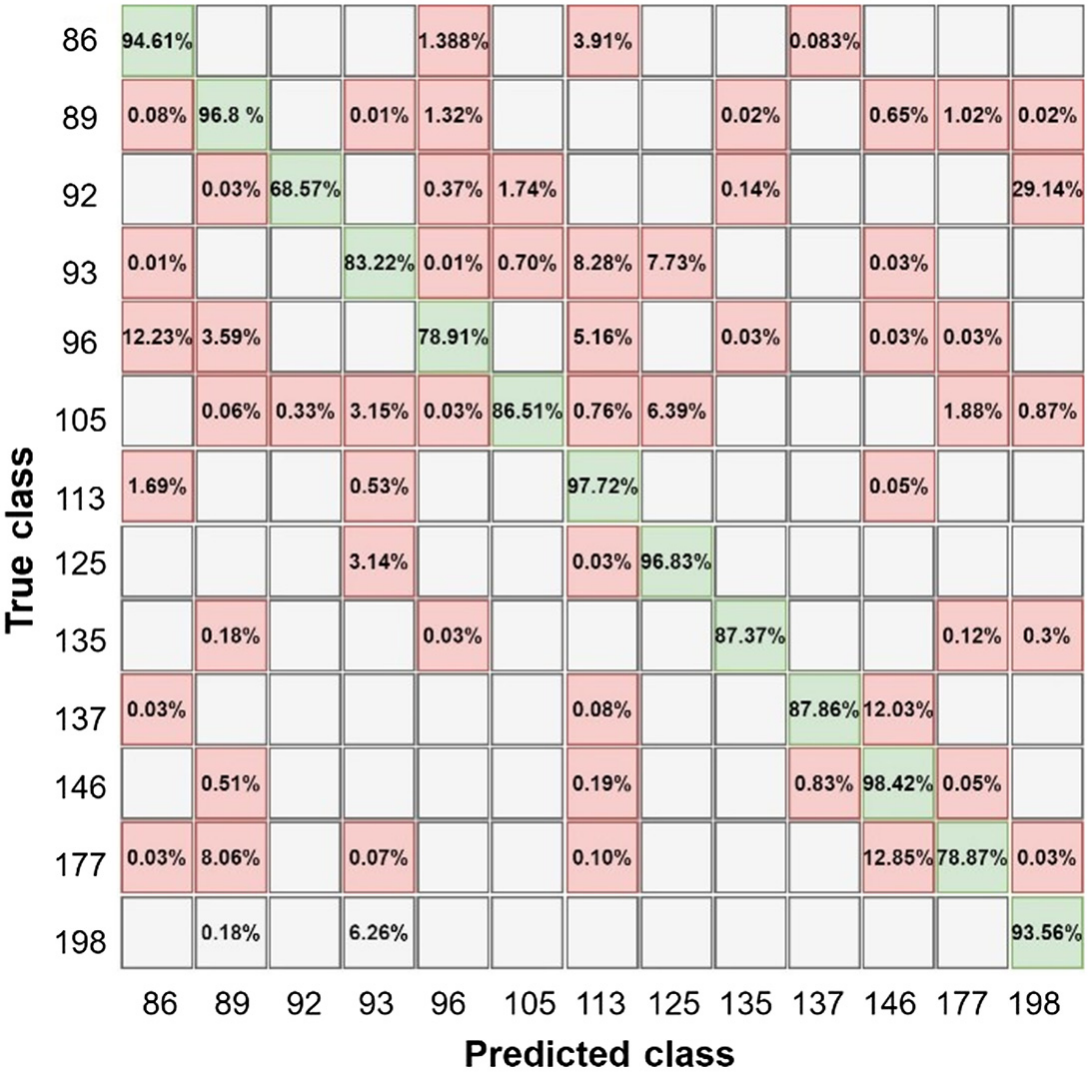
Confusion matrix of CNN testing data.

Additionally, a low degree of false positive (FP) and false negative (FN) rates may be seen in the confusion matrix (CM), demonstrating the model’s validity and capacity to handle different types of data noise. Figure 9 shows the CM determining the model accuracy of the classification results for each glucose level. The number of iterations for training our network is displayed in Fig. 8, representing the longer training duration compared with ML. The training saturation is at a relatively slow stage of the training process, according to an analysis of the network’s performance. The network performance can be enhanced using more convolution layers and advanced dataset analysis to increase the training accuracy.

### 5.2 Evaluating Glucose Detection Accuracy Using ML

The speckle data for the ML algorithm are converted into time series, as explained in Sec. 4. The data under a magnetic field at 140 Hz are filtered, which is highly selective based on different glucose levels. The optimized model increases the algorithm’s ability to classify the testing data. Figure 12 illustrates the evaluation of the SVM classifier, which gives the best accuracy compared with other classification algorithms, as explained in Sec. 4.2. To see how well the classifier performs, we compared the true class labels to the predicted class labels using the confusion matrix. The confusion matrix’s diagonal elements represent the correctly categorized data samples. The matrix helps to visualize the accuracy of data classification for various glucose levels. Table 4 presents the processing results of the ML algorithm used. The table shows the accuracy, precision, sensitivity, and F1 score.

**Table 4:** ML algorithm processing results.

Configurations	Accuracy	Precision	Sensitivity	F1 score
AC-induced magnetic field	97.3%	0.9752	0.9753	0.9660
Normal condition	61.4%	0.6616	0.6394	0.632

**Fig. 10:**
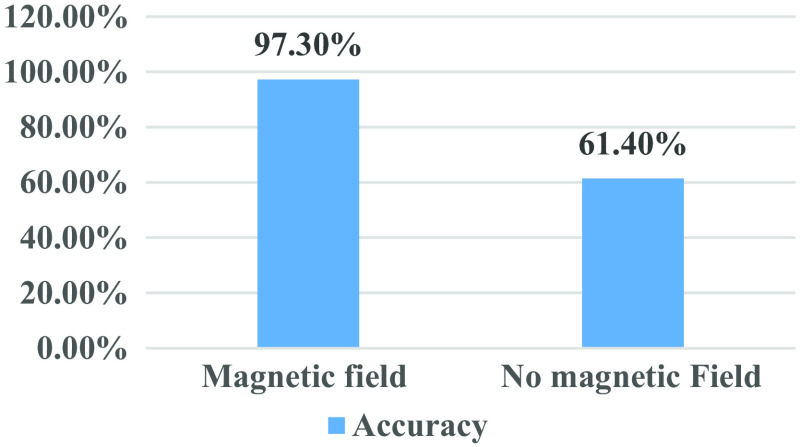
Accuracy of blood glucose classification using SVM.

**Fig. 11:**
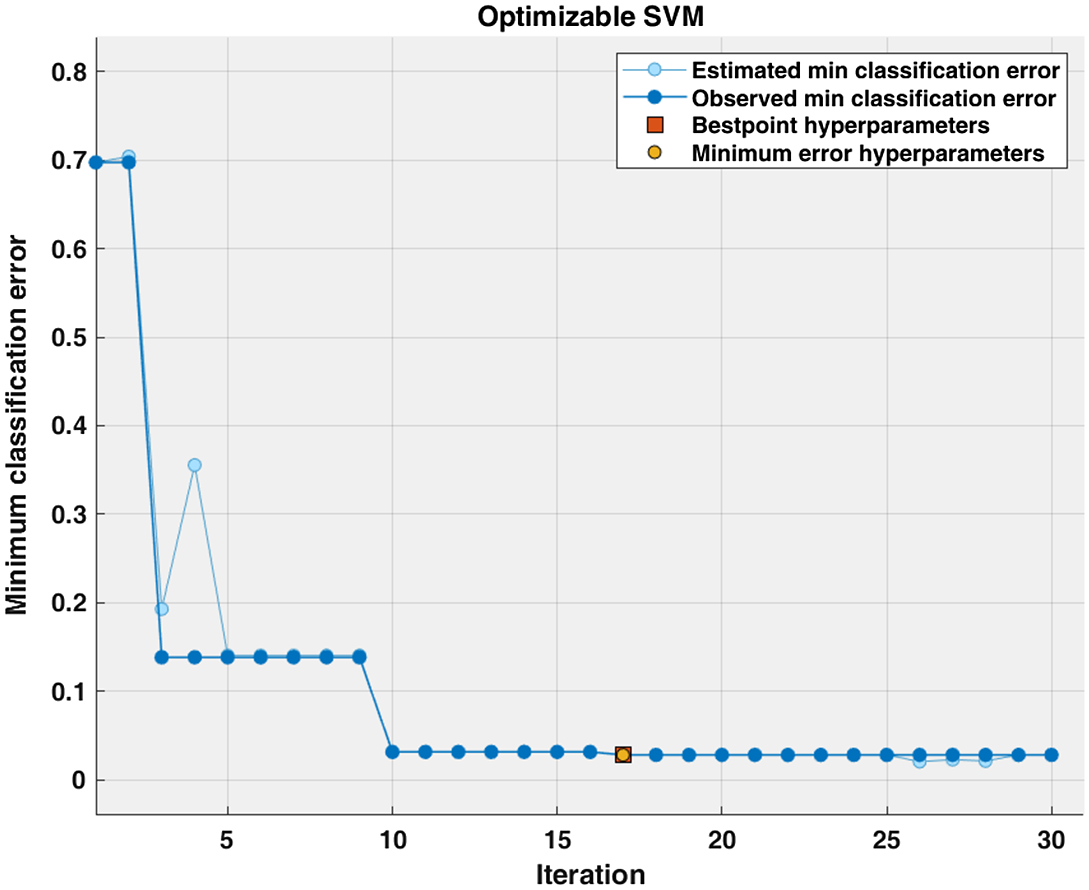
Optimized SVM classification algorithm’s training procedure.

**Fig. 12:**
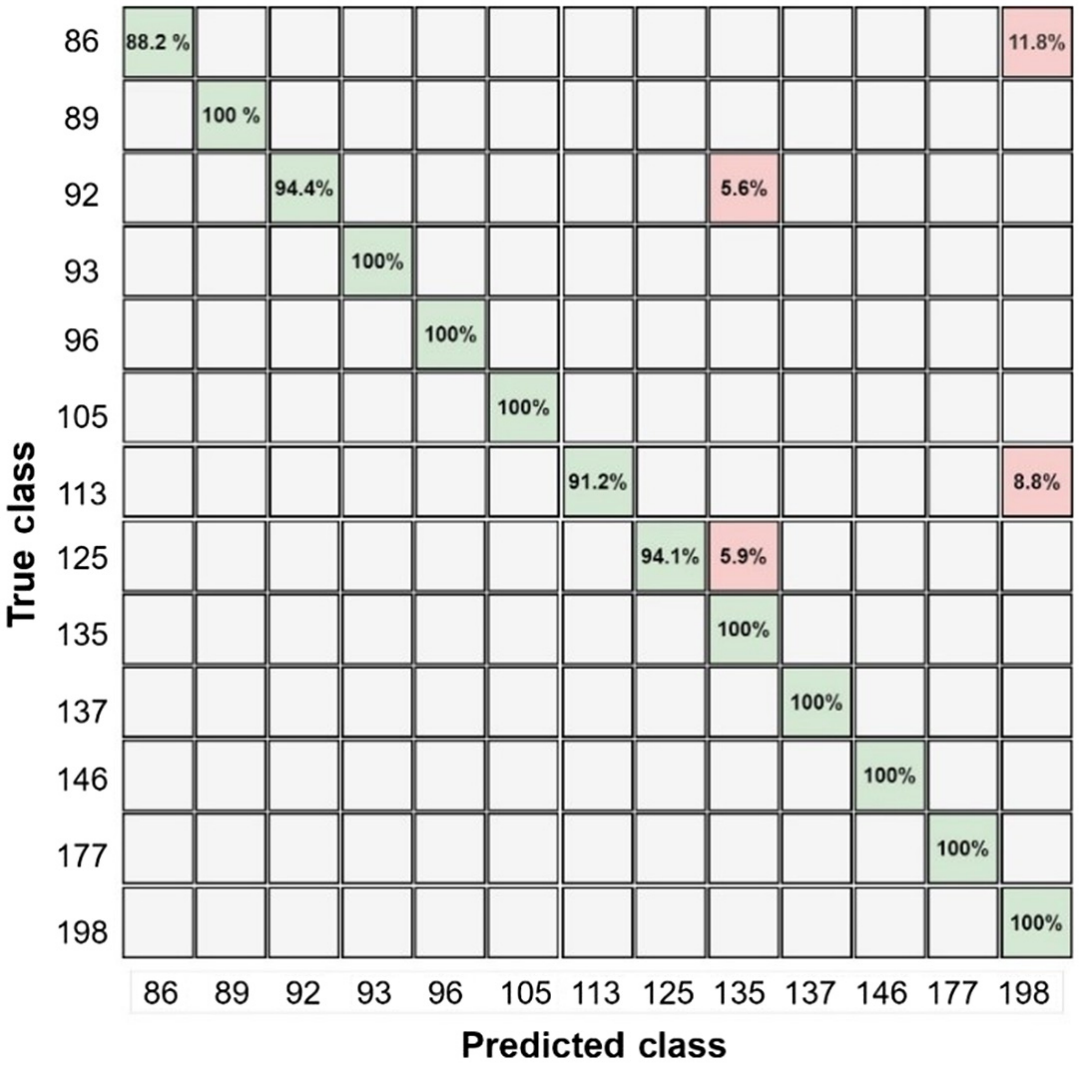
Confusion matrix of SVM algorithm training data.

The accuracy of blood glucose level detection has significantly increased under the AC-induced magnetic field at a lock-in frequency of 140 Hz, as illustrated in Fig. 10. Figure 11 depicts the training procedure for our optimized SVM algorithm representing a very low classification error in significantly fewer iterations compared with CNN. The trained algorithm performance was analyzed using the testing data for blood glucose classification. The confusion matrix shows the classification accuracy of different glucose levels in Fig. 12. Table 4 includes the accuracy of 97.3% for the AC magnetic field derived from the confusion matrix for classifying various glucose levels. The F1 score demonstrates a higher accuracy and ability to identify different glucose levels, with an average F1 score of 0.966.

Additionally, the precision and sensitivity for each glucose class are calculated and averaged. The results are presented in Table 4, indicating the effectiveness of the magneto-optic technique combined with the ML algorithm in accurately classifying blood glucose levels. The magneto-optic effect under the AC-generated magnetic field at a fixed frequency reduces the external noise by being highly selective, thus improving the overall sensitivity for detecting changes in the glucose concentration.

## 6 Discussion and Conclusion

This work presented a novel method for noninvasive human blood glucose sensing using an indirect approach that extracts the blood glucose level from reflected laser speckle pattern recordings taken under normal conditions and under an AC magnetic field inferred at a fixed frequency to develop the magneto-optic effect. The system consists of a laser source to illuminate the finger skin, a digital camera that recorded the patterns of reflected speckles, a magnetic field inductor, and a computer with Matlab. The magneto optic effect showed an improvement in blood glucose level identification.

The use of advanced AI methods DNN and machine learning (ML) techniques allowed for the classification of blood glucose levels with high precision. The classification models were optimized after selecting the best hyperparameter value to predict glucose levels. The optimized model gives improved and accurate results. We designed a CNN model adapted to analyze image data to perform the classification from the direct secondary speckle patterns analysis. In ML, the speckle pattern data were modified to the temporal shift in 2D time series data before the model application.

Experimental result have shown the viability of remote blood glucose levels classification by analyzing laser-induced speckle patterns reflected from human skin while being subjected to an AC magnetic field. The choice of the AC frequency is crucial in this method. Following preliminary experiments at various operating frequencies (60, 90, and 140 Hz), the 140 Hz frequency was chosen as it produces better results by maintaining the maximum stable magnetic field of 150 Gauss throughout the experiment. Increasing the magnetic field strength can further enhance the accuracy of the detection. However, due to the limitation of the setup, we kept the magnetic field strength at 150 Gauss. The direct approach of the finding glucose concentration from the recorded speckle pattern using the magneto-optic effect enhances the highest detection accuracy of 97.3% in the ML case due to the preprocessing of acquired data, which is highly selective at the inferred frequency. In the future, the AC-induced magnetic field inferred at an optimized frequency can be extracted more efficiently using advanced image feature extraction techniques.

The accuracy of the DNN model, which currently stands at around 90% has the potential for further improvement. This is because the speckle pattern images contain more information compared to the 2D data used in ML model, leading to a partial data loss. A large-scale sampling study will allow for further improvement of blood glucose classification accuracy.

This study presents a promising approach for noninvasive blood glucose monitoring using a magneto-optic effect and AI techniques. Our optical system’s sensitivity was determined by the ratio of the change in sensor output to glucose concentration, and we achieved a sensitivity of 1  mg/dl for glucose levels ranging from 86 to 198  mg/dl with a sampling volume of 2501 frames taken under a sampling rate of 500 FPS. However, it is difficult to directly compare the sensitivity of the different methods shown in Table 1 because they have different measurement principles and units. Further investigation is required to validate our results, especially in terms of the limit of detection and optimal sampling rate of our technique. We believe that, with improvements in the experimental setup, building a bracelet based prototype connected to a smartphone, and the use of more advanced AI methods, the accuracy and feasibility of our proposed approach for onsite or online monitoring of blood glucose levels can be enhanced. Overall, our results demonstrate the potential of our optical system for accurate and noninvasive glucose level monitoring, which can have significant implications for the management of diabetes and other related conditions.
